# Supplementary material for: Content-rich biological network constructed by mining PubMed abstracts
Source: BMC Bioinformatics. 2004 Oct 8;5:147. doi: 10.1186/1471-2105-5-147 (PMC528731; doi:10.1186/1471-2105-5-147)
Supplement: Additional File 2 — The original results of the above study (non-essential files are deleted to keep the file size under the limit set by BMC bioinformatics). [file 1471-2105-5-147-S2.bz2 › chilibotAdditionalFile2/dip50/304ID9325095E1301/html/APP_ACHE.html]

 


 **APP** and **ACHE** 
  
Found 98 abstracts in PubMed, retrieved 50.  
 

 What does Google say? 
 PDF only 
| .edu only 

---

**Interactive relationship** (e.g. stimulation, inhibition, etc)

**Inhibitory relationship**- These results indicate that the inhibitory potencies of the active site anti ChE agents on the AAA  [ **APP** ]  activity associated with eel  **AChE**  and horse serum BuChE are closely correlated with their action on the respective ChE.  Ref: 9634011 Biochem Pharmacol, 1998
- The results suggest that  **AChE**  inhibitors modulate the metabolism of  **APP** , possibly via their stimulatory effects on PKC.  Ref: 10773217 Brain Res, 2000
- The results suggest that these  **AChE**  inhibitors promote the non amyloidogenic route of  **APP**  processing, which may be due to their stimulatory effects on PKC.  Ref: 11099780 Neurochem Int, 2001
- Tolerance to cholinesterase inhibition might be induced in the mouse brain because the inhibition rate of  **AChE**  was attenuated from about 80 to 50% during the experiment in both  **APP**  PS1 and nontransgenic mice.  Ref: 11822883 Exp Neurol, 2002
- We screened 42 Korean traditional tea plants to determine the inhibitory effect of acetylcholinesterase  [ **ACHE** ]  and attenuation of toxicity induced by amyloid beta  [ **APP** ]  peptide, which were related to the treatment of Alzheimer s disease AD.  Ref: 10901162 Mol Cells, 2000
- Alterations in  **APP**  metabolism have recently been proposed to play a key role in the long lasting effects of  **AChE**  inhibitors.  Ref: 12769797 Curr Drug Target CNS Neurol Disord, 2003
- It has been suggested that acetylcholinesterase  [ **ACHE** ]   **AChE**  inhibitors, which promote the cholinergic function and consequently improve the cognitive deficits, may also exert a neuroprotective effect by activating normal  **APP**  processing.  Ref: 10773217 Brain Res, 2000
**Stimulatory relationship**- Amyloid beta  [ **APP** ]  increases acetylcholinesterase  [ **ACHE** ]  expression in neuroblastoma cells by reducing enzyme degradation.  Ref: 12871588 J Neurochem, 2003
- Levels of salt extractable  **AChE**  isoforms were increased in the brains of  **APP**  CT100 mice.  Ref: 9681463 J Neurochem, 1998
- Involvement of oxidative stress in the enhancement of acetylcholinesterase  [ **ACHE** ]  activity induced by amyloid beta  [ **APP** ]  peptide.  Ref: 12507730 Neurosci Res, 2003
- In particular, it appears that  **AChE**  may directly interact with amyloid beta  [ **APP** ]  in a manner that increases the deposition of this peptide into insoluble plaques.  Ref: 12669110 Drugs Today Barc , 2003
- We have previously shown that  **AChE**  expression in P19 cells is increased by the amyloid beta  [ **APP** ]  protein.  Ref: 9681463 J Neurochem, 1998
- We now report that an irreversible  **AChE**  inhibitor metrifonate increase the cell associated  **APP**  level in a basal forebrain neuronal culture and also elevate the amount of  **APP**  secreted into the medium.  Ref: 10773217 Brain Res, 2000
**Neutral relationship**- Acetylcholinesterase  [ **ACHE** ]  amyloid beta  [ **APP** ]  peptide interaction and Wnt signaling involvement in Abeta neurotoxicity.  Ref: 11261806 Acta Neurol Scand Suppl, 2000
- A structural motif of acetylcholinesterase  [ **ACHE** ]  that promotes amyloid beta  [ **APP** ]  peptide fibril formation.  Ref: 11523986 Biochemistry, 2001
- We have shown that  **AChE**  interacts with the amyloid beta  [ **APP** ]  peptide Abeta and promotes amyloid fibril formation by a hydrophobic environment close to the peripheral anionic binding site PAS of the enzyme.  Ref: 11523986 Biochemistry, 2001
- Analysis of  **AChE**  by lectin binding revealed differences in the glycosylation pattern in  **APP**  CT100 mice similar to those observed in frontal cortex samples from AD.  Ref: 9681463 J Neurochem, 1998
- Amyloid fibril formation by a synthetic peptide from a region of human acetylcholinesterase  [ **ACHE** ]  that is homologous to the Alzheimer s amyloid beta  [ **APP** ]  peptide.  Ref: 12427014 Biochemistry, 2002
- Neurotoxicity of acetylcholinesterase  [ **ACHE** ]  amyloid beta  [ **APP** ]  peptide aggregates is dependent on the type of Abeta peptide and the  **AChE**  concentration present in the complexes.  Ref: 10359075 FEBS Lett, 1999
- A region near the C terminus of human acetylcholinesterase  [ **ACHE** ]   **AChE**  is weakly homologous with the N terminus of the Alzheimer s disease amyloid beta  [ **APP** ]  peptide.  Ref: 12427014 Biochemistry, 2002
- The results are consistent with the possibility that changes in  **AChE**  isoform levels and glycosylation patterns in the AD brain may be a direct consequence of altered  **APP**  metabolism.  Ref: 9681463 J Neurochem, 1998
- CONCLUSIONS Administration of  **AChE**  inhibitors increases the ratio of  **APP**  forms in platelets of patients with AD, suggesting a potential effect of  **AChE**  inhibitors on  **APP**  trafficking or processing in a peripheral cell.  Ref: 11255448 Arch Neurol, 2001
- However, the presence of both 1% Triton X 100 and paraoxon at concentrations of 5 nM 100 nM resulted in complex kinetic interactions with acetylcholinesterase  [ **ACHE** ] , as evidenced by a curvilinear secondary plot for determination of the  **app**  k i.  Ref: 11568364 Toxicol Sci, 2001
- Acetylcholinesterase  [ **ACHE** ]   **AChE**  and butyrylcholinesterase BChE from vertebrates, other than their predominant acylcholine hydrolase esterase activity, display a genuine aryl acylamidase activity AAA  [ **APP** ]  capable of hydrolyzing the synthetic substrate o nitroacetanilide to o nitroaniline.  Ref: 11034785 BIO ORGANIC CHEMISTRY< MedlineTA>BIO ORGANIC CHEMISTRY, 2001

**Non-interactive relationship** (e.g. studied together, co-existance, homology, etc.)

- This study examined the question of whether acetylcholinesterase  [ **ACHE** ]   **AChE**  inhibitors can alter the processing of  **APP**  and the level of protein kinase C PKC in primary rat basal forebrain cultures.  Ref: 11099780 Neurochem Int, 2001
- Further work is warranted to elucidate the exact roles in  **APP**  metabolism of the  **AChE**  inhibitors used in AD therapy at present.  Ref: 12769797 Curr Drug Target CNS Neurol Disord, 2003
- Senile plaques are mainly composed of amyloid beta  [ **APP** ]  peptide Abeta fibrils and several proteins including acetylcholinesterase  [ **ACHE** ]   **AChE** .  Ref: 10359075 FEBS Lett, 1999
- This review surveys recent data from in vivo and in vitro studies that have contributed to our understanding of the role of  **AChE**  inhibitors in  **APP**  processing.  Ref: 12769797 Curr Drug Target CNS Neurol Disord, 2003
- Western blotting was used to test two  **AChE**  inhibitors reversible and irreversible for their ability to enhance the release of  **APP**  and PKC content.  Ref: 11099780 Neurochem Int, 2001
- Altered glycosylation of acetylcholinesterase  [ **ACHE** ]  in  **APP**  SW Tg2576 transgenic mice occurs prior to amyloid plaque deposition.  Ref: 12065653 J Neurochem, 2002
- Previous studies have indicated that acetylcholinesterase  [ **ACHE** ]   **AChE**  promotes amyloid beta  [ **APP** ]  peptide Abeta fibril formation and  **AChE**  Abeta complexes increase Abeta dependent neurotoxicity.  Ref: 11261806 Acta Neurol Scand Suppl, 2000
- There was also an increase in amphiphilic monomeric form of  **AChE**  in the  **APP**  CT100 mice.  Ref: 9681463 J Neurochem, 1998
- OBJECTIVE To investigate whether acetylcholinesterase  [ **ACHE** ]   **AChE**  inhibitor treatment modifies the ratio of platelet  **APP**  forms in patients with AD.  Ref: 11255448 Arch Neurol, 2001
- The rank order of the same compounds as  **AChE**  inhibitors basically paralleled that of AAA  [ **APP** ] , although they were in general stronger on  **AChE**  Ki = 0.001 0.05.  Ref: 9634011 Biochem Pharmacol, 1998
- Neostigmine, physostigmine, BW 284C51, huperzine A, E2020, tacrine, edrophonium and heptyl physostigmine were, in that order, the most potent in inhibiting eel  **AChE**  associated AAA  [ **APP** ]  activity, their inhibitor constant Ki values being in the range 0.02 0.37 microM.  Ref: 9634011 Biochem Pharmacol, 1998
- Based on the data of this study, we concluded that the number of intrinsic cardiac neurons may be approximated  [ **APP** ]  in the total heart preparation via counting and measuring of intramural ganglia, contours of which are well discernible following a histochemical reaction for  **AChE** .  Ref: 11936191 Ann Anat, 2002
- **APP**  gene expression was unaffected by long term  **AChE**  inhibition suggesting that increased short term sAPPalpha release does not elicit compensatory effects.  Ref: 11526466 Mol Psychiatry, 2001
- The senile plaques present in Alzheimer s disease AD are composed of a core of amyloid beta  [ **APP** ]  peptide Abeta plus several proteins including acetylcholinesterase  [ **ACHE** ]   **AChE** .  Ref: 9883888 FEBS Lett, 1998
- In this study we report that amyloid beta  [ **APP** ]  peptides Abeta increased  **AChE**  levels in both neuronal and astrocytic primary cultures, supporting the possibility that both neurons and glia may make a direct contribution to the pool of  **AChE**  seen around amyloid deposits in the AD brain.  Ref: 12591148 Brain Res, 2003
- Glyc  **AChE**  is increased in  **APP**  SW Tg2576 transgenic mice prior to amyloid plaque deposition, which suggests that Glyc  **AChE**  may be an early marker of AD.  Ref: 12704813 J Neurosci Res, 2003
- We have investigated the acute and chronic effect of metrifonate MTF and dichlorvos DDVP, respectively the prodrug and active acetylcholinesterase  [ **ACHE** ]  inhibitor, on the secretory processing of the amyloid precursor protein  **APP**  in SH SY5Y neuroblastoma cells.  Ref: 11526466 Mol Psychiatry, 2001
- The peripheral anionic site inhibitors propidium and gallamine were inactive on  **AChE**  associated AAA  [ **APP** ] .  Ref: 9634011 Biochem Pharmacol, 1998
- The nicotinic nAChRs and muscarinic mAChRs acetylcholine receptors and acetylcholinesterase  [ **ACHE** ]   **AChE**  activity were studied in the brains of  **APP**  SWE transgenic mice Tg and age matched nontransgenic controls Tg that were between 4 and 19 months of age.  Ref: 12093166 Mol Cell Neurosci, 2002
- To investigate whether acetylcholinesterase  [ **ACHE** ]  inhibitors ChEIs could decrease production of amyloid beta  [ **APP** ]  peptide A beta and slow down the accumulation of A beta also in vivo, we chronically administered metrifonate 100 mg kg, po, a second generation ChEI, to 7 month old doubly transgenic  **APP**  PS1 mice and their nontransgenic littermate controls for 7 months.  Ref: 11822883 Exp Neurol, 2002
- Since both a decreased level of  **APP**  secretion and a low activity and level of PKC may be involved in the pathogenesis of AD, it is concluded that the administration of  **AChE**  inhibitors to AD patients may facilitate the memory processes and exert a neuroprotective effect.  Ref: 11099780 Neurochem Int, 2001
- The potency of a series of anticholinesterase anti ChE agents and serotonin related amines as inhibitors of the aryl acylamidase AAA  [ **APP** ]  activity associated with electric eel acetylcholinesterase  [ **ACHE** ]   **AChE**  EC 3.1.1.7 and horse serum butyrylcholinesterase BuChE EC 3.1.1.8 was examined and compared with the potency of the same compounds as ChE inhibitors.  Ref: 9634011 Biochem Pharmacol, 1998
- Serotonin and its derivatives were slightly stronger on AAA  [ **APP** ]  Ki = 7.5 30 microM than on  **AChE**  Ki = 20 140 microM.  Ref: 9634011 Biochem Pharmacol, 1998
